# A hierarchical integration deep flexible neural forest framework for cancer subtype classification by integrating multi-omics data

**DOI:** 10.1186/s12859-019-3116-7

**Published:** 2019-10-28

**Authors:** Jing Xu, Peng Wu, Yuehui Chen, Qingfang Meng, Hussain Dawood, Hassan Dawood

**Affiliations:** 1 School of Information Science and Engineering, University of Jinan, Jinan, China; 2 Shandong Provincial Key Laboratory of Network Based Intelligent Computing, Jinan, China; 3 Department of Computer and Network Engineering, University of Jeddah, Jeddah, Saudi Arabia; 4 Department of Software Engineering, University of Engineering and Technology, Taxila, Pakistan

**Keywords:** Autoencoder, Cancer subtype classification, Cascade forest, Data integration, Deep learning

## Abstract

**Background:**

Cancer subtype classification attains the great importance for accurate diagnosis and personalized treatment of cancer. Latest developments in high-throughput sequencing technologies have rapidly produced multi-omics data of the same cancer sample. Many computational methods have been proposed to classify cancer subtypes, however most of them generate the model by only employing gene expression data. It has been shown that integration of multi-omics data contributes to cancer subtype classification.

**Results:**

A new hierarchical integration deep flexible neural forest framework is proposed to integrate multi-omics data for cancer subtype classification named as HI-DFNForest. Stacked autoencoder (SAE) is used to learn high-level representations in each omics data, then the complex representations are learned by integrating all learned representations into a layer of autoencoder. Final learned data representations (from the stacked autoencoder) are used to classify patients into different cancer subtypes using deep flexible neural forest (DFNForest) model.Cancer subtype classification is verified on BRCA, GBM and OV data sets from TCGA by integrating gene expression, miRNA expression and DNA methylation data. These results demonstrated that integrating multiple omics data improves the accuracy of cancer subtype classification than only using gene expression data and the proposed framework has achieved better performance compared with other conventional methods.

**Conclusion:**

The new hierarchical integration deep flexible neural forest framework(HI-DFNForest) is an effective method to integrate multi-omics data to classify cancer subtypes.

## Background

Cancers are considered as heterogeneous disease including the multiple pathogenesis and clinical features [1, 2]. Cancers have been divided into several subtypes based on different outcomes and treatments. Cancer subtype classification can provide a detailed understanding into cancer pathogenesis which helps to diagnose the cancer accurately and personalized cancer treatment [3]. Cancer subtypes classification has been widely studied over the last decade [4–8]. It has been shown that different subtypes are usually caused by different genetic mutations [9–11].

Recent advancements of high-throughput sequencing technology have enabled us to collect multi-omics data from the same cancer samples, such as gene expression, miRNA expression and DNA methylation data. The Cancer Genome Atlas (TCGA) [12, 13] project produced different kinds of genome, transcriptome and epigenome information for more than 1100 patient samples from more than 34 cancer types [14]. These sequencing data provide an unprecedented opportunity to study cancer subtype at the molecular level by using multi-omics data [15, 16]. Many computational methods have been proposed to classify cancer subtypes [17–21], however most of them generate the model by only employing gene expression data. It has been shown that integration of multi-omics data provides better cancer subtype classification in recent years [22–24]. The miRNA plays an important role in cancer progression by complementing mRNA and in mRNA silencing or degradation [25–28]. DNA methylation is a chemical modification of DNA that can change genetic performance without changing the DNA sequence. Thus, there is a need for a computational approach that enables the comprehensive analysis of these multi-omics data as well as reliable integration of information generated from different platforms.

The simplest way to combine biological data is to connect standardized measurements from a variety of biological fields, such as miRNA expression and DNA methylation, however the results are not ideal. A common strategy is to analyze each data type independently [29–32] and combine the data. That often leads to unreliable conclusions that makes it difficult to integrate. Moreover, analysis of this cross-platform genomic data also poses new challenges for traditional data analysis methods [33, 34], such as K-means clustering method [35] or principal component analysis (PCA) [36]. Usually, multi-omics data come from multiple platforms, which typically have different representations and statistical properties. Also, multi-omics data for the same cancer is unlikely to be independent. In order to solve this problem, we propose a hierarchical integration stacked autoencoder, taking both the intrinsic statistical properties of each individual types of data and the correlation of different omics data into account.

Biological data typically have high dimensionality and small sample sizes, which poses great challenge to traditional classification methods. With the rapid development in machine learning techniques [37, 38], particularly in deep learning which allowed direct processing of such high dimensional biological data without knowing the prior knowledge. The performance of deep neural networks (DNN) mainly depends on its structure, however no effective structural optimization algorithms have been proposed. Deep forest [39] was proposed as an alternative to solve the structural design problems of DNN. Inspired by deep forest, deep flexible neural forest (DFNForest) was proposed, which is an ensemble of flexible neural tree (FNT) [40, 41]. DFNForest overcomes the problem of increasing the depth of FNT and dealing with multi-classification.

In this paper, a hierarchical integration deep flexible neural forest (HI-DFNForest) framework has been proposed to integrate multi-omics data for cancer subtype classification. We integrated gene expression, miRNA expression and DNA methylation data with stacked autoencoder [42, 43] for cancer subtype classification. Specifically, we propose to use stacked autoencoders to learn the representations of each omics data. Secondly, an autoencoder is used to learn complex representations according to the learned features. Finally, previously learned complex representation is used as input to the DFNForest model for cancer subtype classification. The entire process is called HI-DFNForest framework.

The main contributions are summarized below.

(1) Integration of gene expression, miRNA expression and DNA methylation data, which offers more comprehensive prospects for cancer subtype classification. Most of the current cancer subtype classification methods are based on gene expression data. In fact, miRNA expression and DNA methylation are also closely related to abnormal gene mutations in cancer.

(2) Proposal of a hierarchical integration stacked autoencoder which takes the intrinsic statistical properties of individual types of data and the correlation of different omics data into account. A high-level representation in each omics data is learned separately using a stacked autoencoder (SAE) and all learned representations are integrated into an autoencoder to learn complex data representations.

(3) Proposal of a hierarchical integration deep flexible neural forest (HI-DFN Forest) framework to integrate multi-omics data for cancer subtype classification. Hierarchical stacked autoencoder is used to learn high-level features from each omics data, then the final integrative data representations are used to classify patients into different cancer subtypes using DFNForest model.

## Results

### Datasets

To show the effectiveness of HI-DFNForest framework, three different cancer types from the TCGA [12, 13] are considered. The three cancer types include breast invasive carcinoma (BRCA) with 104 samples, glioblastoma multiforme (GBM) with 213 samples and ovarian cancer (OV) with 102 samples. For each of cancers, Level 3 dataset containing gene expression, miRNA expression and DNA methylation data are used. Before applying our HI-DFNForest framework, we performed three steps of pre-processing: outlier deletion, missing data imputation, and normalization [44]. If a biological feature has more than 20% missing values in a patient, this patient data is filtered out. In addition, for missing data, we use K nearest neighbor (KNN) for imputation. Finally, before classifying cancer subtypes, we performed the following normalization:

1 $$\begin{array}{@{}rcl@{}} \widetilde{f}=\frac{f-E(f)}{\sqrt{Var(f)}} \end{array} $$

Where *f* is any biological feature, $\widetilde {f}$ is the corresponding features after normalization, *E*(*f*) and *Var*(*f*) are the mean and variance of *f*.

Table 1 shows the details of datasets. We also downloaded the corresponding clinical data for each data set from TCGA to label each sample.

**Table 1:** Statistics of datasets for three cancer types

Cancer type	DNA methylation	miRNA expression	Gene expression	Patient
BRCA	23094	354	17814	104
GBM	1305	534	12042	213
OV	24963	539	16860	102

### Model selection

There are three different forests developed for the experiment in HI-DFNForest model. For the three forests, the function set F was set to {+_2_,+_3_, +_4_}, {+_2_,+_4_, +_5_},{+_3_,+_4_, +_5_} respectively. As for the base classifier FNT, its structure is optimized by grammar guided genetic programming and parameters is optimized by particle swarm optimization. 5-fold cross-validation is used to assess the performance of different parameter settings in FNT, the smallest root mean square error(RMSE) can be obtained, and the corresponding parameter settings of FNT are shown in Table 2.

**Table 2:** Parameter settings of FNT

Parameter	Value
Population size	50
Crossover probability	0.4
Mutation probability	0.01
C1	2
C2	2
Vmax	2

In order to choose a better SAE structure, we trained the SAE according to the different number of hidden layers and hidden variables, which is compared on the mean square error (MSE) value. Different structures of SAE have been considered and best one is chosen as the parameter of model. For gene expression data and DNA methylation, the best structure was a three-layer SAE of 500-200-50. For miRNA expression, the smallest MSE of structure was a two-layer SAE, and the number of hidden variables was 100-50. The last level is the AE of 50 hidden variables.

To check whether the HI-DFNForest model is overfitting, the permuted input data set is used as input to the proposed model, and the experimental results are compared with random guess. The experiments are randomly performed 10 times, and the average of the results is compared with the accuracy of the random guess. The input data used are BRCA data set, because it is a classification problem of 4 cancer subtypes, so the accuracy of random guess is 0.25. The result of the permuted input data set is 0.484, which is higher than the accuracy of random guess. The reason why the accuracy of HI-DFNForest is higher than that of random guessing is that the proposed model has a training process, but random guessing does not have this process. Therefore, the classification performance of HI-DFNForest is not significantly higher than a random guess, indicating that our model is not overfitting. The main reasons why the HI-DFNForest model is not overfitting are: (1) the base classifier FNT is a sparse structure that allows cross-layer connections, which avoids overfitting and has good generalization performance. (2) the proposed model adopts a cascade structure, and the level of the cascade structure is adaptively determined. When the accuracy does not change on the validation set, the number of levels does not increase, so it is suitable for small-scale data.

### Comparison of proposed method with multiple and single dimensional data

To test whether integration of multi-omics data contributes to cancer subtype classification, we used data from DNA methylation, miRNA expression, gene expression and integration of these three types of data using SAE as input to our DFNForest classification model, respectively. On the breast invasive carcinoma (BRCA), glioblastoma multiforme (GBM) and ovarian cancer (OV) data sets, using the classification accuracy as the basis for evaluating the performance.

**Fig. 1:**
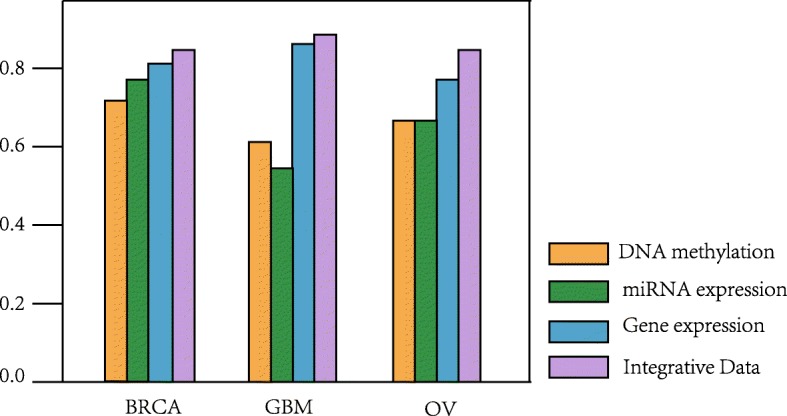
Comparison of classification accuracy between different data

As shown in Table 3, it is clear that the performance of integrative data is superior to using only DNA methylation, miRNA expression, and gene expression data. For example, in the BRCA data set, the classification accuracy rate of integrative data set reaches 0.846, while the accuracy of DNA methylation is 0.731, the accuracy of miRNA expression is 0.769, and the accuracy of gene expression is 0.808. Meanwhile, in the GBM data set, the classification accuracy rate of integrative data set reaches 0.885, while the accuracy of DNA methylation is 0.596, the accuracy of miRNA expression is 0.539, and the accuracy of gene expression is 0.865. In the OV data set, the classification accuracy rate of integrative data set reaches 0.840, while the accuracy of DNA methylation is 0.640, the accuracy of miRNA expression is 0.640, and the accuracy of gene expression is 0.760. Table 3 demonstrates that integrative data improves classification accuracy compared to only using one omics data as input. Figure 1 shows the classification results of different omics data. As can be seen from Fig. 1, when gene expression data and integration data are used as inputs, the accuracy is higher, however, DNA methylation and miRNA expression are less accurate. The main purpose of proposed HI-DFNForest framework is to use DNA methylation and miRNA expression as supplementary information for gene expression in cancer subtype classification. The experiments prove that the proposed integration method has improved the performance as compared to only using gene expression data.

**Table 3:** Performance comparison of the proposed method with multiple and single dimensional data

Cancer type	DNA methylation	miRNA expression	Gene expression	Integrative Data
BRCA	0.731	0.769	0.808	0.846
GBM	0.596	0.539	0.865	0.885
OV	0.640	0.640	0.760	0.840

### Comparison with other dimensionality reduction methods

In HI-DFNForest, a hierarchical integration SAE framework is used to learn the representation of input data. To assess the performance of SAE in learning features, comparing with traditional principal components analysis (PCA) and non-negative matrix factorization (NMF) methods using DNA methylation, miRNA expression, gene expression and integrative data on BRCA, GBM and OV dataset. Classification accuracy is used as a criterion for judging the learning features of these three dimensionality reduction methods.

**Fig. 2:**
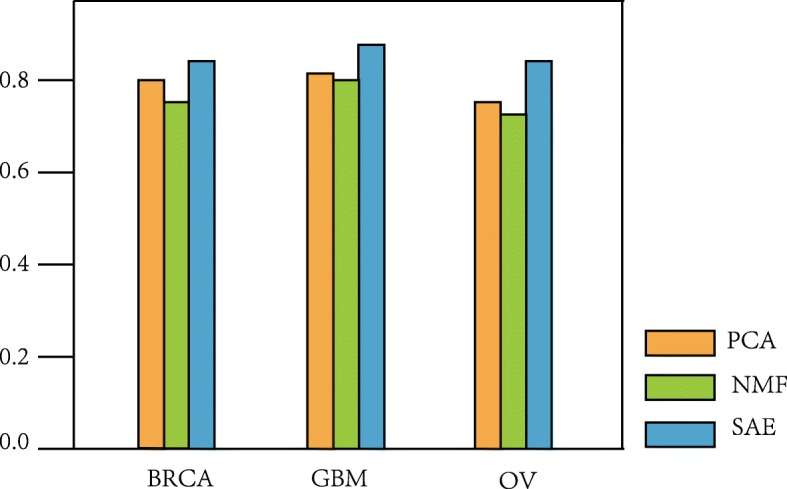
Performance comparison of proposed SAE framework, PCA and NMF using integrative data

Tables 4, 5 and 6 show the comparison of our SAE dimensionality reduction method with PCA and NMF on BRCA, GBM and OV data sets, respectively. The accuracy of our SAE this kind of deep learning model is significantly higher than the traditional PCA and NMF methods for different types of data. For example, our SAE has accuracy of 0.731, while PCA is 0.692 and NMF is 0.654 for DNA methylation data on the BRCA dataset in Table 4. Meanwhile, our SAE has accuracy of 0.865, while PCA is 0.808 and NMF is 0.781 for gene expression data on the GBM dataset in Table 5. Furthermore, SAE has accuracy of 0.840, while PCA is 0.760 and NMF is 0.720 for integrative data on the OV dataset in Table 6. We can see that the accuracy of SAE is the highest as compared to the other two methods, which shows that this deep learning model can learn better than original features while reducing the dimension. Figure 2 clearly demonstrated the performance comparison of our proposed SAE framework, PCA and NMF using integrative data on BRCA, GBM and OV datasets. Under the purpose of learning features and performing dimensionality reduction, our SAE has the best performance, followed by NMF and PCA. Therefore, our hierarchical integration SAE method can effectively integrate multi-omics data, which is conducive to the cancer subtype classification.

**Table 4:** Performance comparison of dimensionality reduction methods on BRCA dataset

Data	PCA	NMF	SAE
DNA methylation	0.692	0.654	0.731
miRNA expression	0.731	0.692	0.769
Gene expression	0.769	0.731	0.808
Integrative Data	0.808	0.769	0.846

**Table 5:** Performance comparison of dimensionality reduction methods on GBM dataset

Data	PCA	NMF	SAE
DNA methylation	0.558	0.577	0.596
miRNA expression	0.519	0.500	0.539
Gene expression	0.808	0.781	0.865
Integrative Data	0.827	0.808	0.885

**Table 6:** Performance comparison of dimensionality reduction methods on OV dataset

Data	PCA	NMF	SAE
DNA methylation	0.600	0.560	0.640
miRNA expression	0.560	0.520	0.640
Gene expression	0.720	0.680	0.760
Integrative Data	0.760	0.720	0.840

### Comparison with other classification methods

To evaluate the performance of our proposed framework, we tested four different models, i.e., k-nearest neighbor (KNN), support vector machine (SVM), random forest (RF), and multi-grained cascade forest (gcForest) instead of DFNForest model in our framework. Integrative data processed by stacked autoencoders are as input to KNN, SVM, RF, gcForest and DFNForest classifiers. Moreover, we compare HI-DFNForest model with mixOmics [45] to evaluate the performance of data integration method. The results are measured by classification accuracy. For fairness, 5-fold cross-validation is used to evaluate the performance of the different classifiers.

**Fig. 3:**
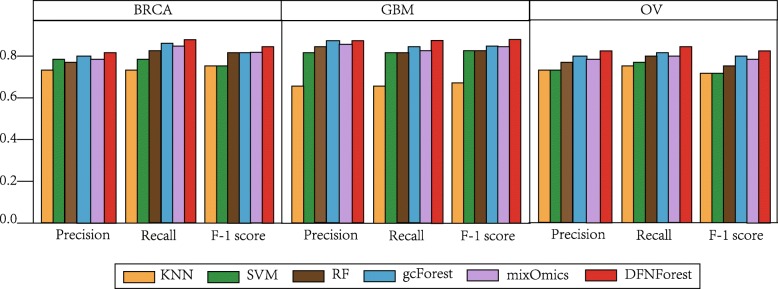
Comparison of overall performance of different classifiers on BRCA, GBM and OV datasets. The average precision, recall and F-1 score of each dataset were evaluated on BRCA, GBM and OV datasets

Tables 7, 8 and 9 show the comparison of DFNForest, KNN, SVM, RF, gcForest and mixOmics on BRCA, GBM and OV data sets, respectively. As we can see, DFNForest has higher classification accuracy than other classifiers. For example, the accuracy of using the integrative data of DFNForest is 0.846, while the KNN is 0.796, the SVM is 0.796, the RF is 0.808, the gcForest is 0.808 and the mixOmics is 0.808 on the BRCA dataset in Table 7. Moreover, the accuracy of using the integrative data of DFNForest is 0.885, while the accuracy of KNN is 0.635, the SVM is 0.846, the RF is 0.846, the gcForest is 0.865 and the mixOmics is 0.846 on the GBM dataset in Table 8. Meanwhile, the accuracy of using the integrative data of DFNForest model is 0.840, while the KNN is 0.720, the SVM is 0.720, the RF is 0.760, the gcForest is 0.800 and the mixOmics is 0.760 in Table 9. It can be observed that most classification methods achieve better performance when using multi-omics data than only using single omics data, which illustrates that DNA methylation and miRNA expression data can be used as complementary information for gene expression data. In addition, compared to the traditional methods like KNN, SVM and RF, gcForest and DFNForest have higher performance because the deep learning models can extract more complex features when processed data layer by layer. However, DFNForest outperforms than gcForest because DFNForest is more applicable to process continuous data. The performance of mixOmics is better than that of traditional methods like KNN, SVM and RF, but worse than DFNForest model. Because mixOmics is a linear model, the performance on such complex multi-omics data is not as good as the proposed HI-DFNForest which is a deep learning model. To assess the overall performance of different classifiers on BRCA, GBM and OV datasets, the average precision, recall and F-1 score of each model were considered. As illustrated in Fig. 3, the DFNForest model has achieved better performance than other methods in cancer subtype classification.

**Table 7:** Comparison of overall accuracy on BRCA datasets

Data	KNN	SVM	RF	gcForest	mixOmics	DFNForest
DNA methylation	0.615	0.692	0.615	0.731	0.692	0.731
miRNA expression	0.654	0.731	0.731	0.731	0.692	0.769
Gene expression	0.731	0.769	0.769	0.769	0.769	0.808
Integrative Data	0.769	0.769	0.808	0.808	0.808	0.846

**Table 8:** Comparison of overall accuracy on GBM datasets

Data	KNN	SVM	RF	gcForest	mixOmics	DFNForest
DNA methylation	0.404	0.558	0.558	0.577	0.558	0.596
miRNA expression	0.539	0.442	0.462	0.558	0.539	0.539
Gene expression	0.635	0.827	0.827	0.846	0.827	0.865
Integrative Data	0.635	0.846	0.846	0.865	0.846	0.885

**Table 9:** Comparison of overall accuracy on OV datasets

Data	KNN	SVM	RF	gcForest	mixOmics	DFNForest
DNA methylation	0.440	0.520	0.560	0.560	0.520	0.640
miRNA expression	0.480	0.520	0.480	0.640	0.560	0.640
Gene expression	0.680	0.680	0.720	0.720	0.720	0.760
Integrative Data	0.720	0.720	0.760	0.800	0.760	0.840

## Discussion

Many computational methods have been proposed to classify cancer subtypes [17–21], however most of them generate the model by only employing gene expression data. Gene expression data is used as input to DFNForest classifier [46]. However, it has been shown that integration of multi-omics data contributes to cancer subtype classification [22–24]. Guo [47] has attempted to use the stacked autoencoder to cluster cancer subtypes, but the difference of our work is that our proposed framework uses three different stacked autoencoders to integrate gene expression, miRNA expression and DNA methylation data, and then using the learned representations as input to the DFNForest model. When gathering multi-omics data, there are usually two main challenges. One is that different input data comes from different platforms, so each type of data has its properties, and the other is that each type of input data cannot be independent. To deal with the above problem, we adopted the hierarchical integration stacked autoencoder. First, the complex features of gene expression, miRNA expression and DNA methylation data are learned by three SAEs with different structures, respectively. After that, the final integrative feature is learned through a layer of AE. Our SAE framework takes both the intrinsic statistical properties of individual types of data and the correlation of different omics data into account. There are some other multi-omics integration methods proposed, such as mixOmics [45]. Although these methods are simpler and easier to implement, they are linear computational models, so the processing performance on complex multi-omics data is not as good as the deep learning model we proposed.

The characteristics of biological data are high dimensionality and small sample sizes, which poses great challenge to traditional classification methods. Recent advances in deep learning have allowed direct processing of such high dimensional data. However, the performance of deep neural networks depends largely on its structure, but no effective structural optimization algorithms have been proposed, usually depending on the individual experience of the researcher. DFNForest was proposed as an alternative to neural networks, which solves structural design problems. Therefore, we design a hierarchical integration deep flexible neural forest framework based on the SAE and DFNForest to integrate multi-omics data to classify cancer subtypes.

Test results on the BRCA, GBM and OV datasets demonstrate that the integration of gene expression, miRNA expression and DNA methylation data have better performance as compared to only using gene expression data, which indicates that DNA methylation and miRNA expression can be used as complementary information for gene expression data in cancer subtype classification. Furthermore, the HI-DFNForest framework can not only integrate different omics data well but also can achieve good classification performance, which may be that our SAE can obtain a better high-level representation of raw data and DFNForest is more applicable to process biological data. In conclusion, the purpose of our HI-DFNForest framework is a new data integration model. Although our HI-DFNForest framework is used to integrate different omics data for cancer subtype classification, it can also be applied to other types of data from different platforms that need to be integrated.

## Conclusions

It is of importance to classify cancer subtypes to promote accurate cancer diagnosis and personalized treatment. Due to the heterogeneity of cancer, it has been proved that integration of multi-omics data has an effect on cancer subtype classification. A deep flexible neural forest framework is proposed to integrate different omics data for cancer subtype classification. Cancer subtype classification is verified on BRCA, GBM and OV data sets from TCGA by integrating gene expression, miRNA expression and DNA methylation data. The autoencoder are stacked to learn data representations from each omics data, then the learned representations are integrated into another autoencoder to learn complex representations. The complex representations that are ultimately learned are used as the input to DFNForest model to classify cancer subtypes. Experiments have shown that integrating multiple omics data improves the accuracy of cancer subtype classification than only using gene expression data, and other omics data can be used as complementary information for gene expression data. Moreover, SAE is actually a dimensionality reduction approach, so we compared it with traditional PCA and NMF methods. The results show our SAE model can better learn the original features and reduce the dimensionality. In addition, the DFNForest model has higher performance compared to the other classifiers. In conclusion, our HI-DFNForest framework based on hierarchical integration stacked autoencoders and DFNForest model provides an option to integrate multi-omics data in the cancer subtype classification.

## Methods

In this section, the stacked autoencoder, deep flexible neural forest and lastly proposed hierarchical integration deep flexible neural forest framework are explained.

### Stacked autoencoder

**Fig. 4:**
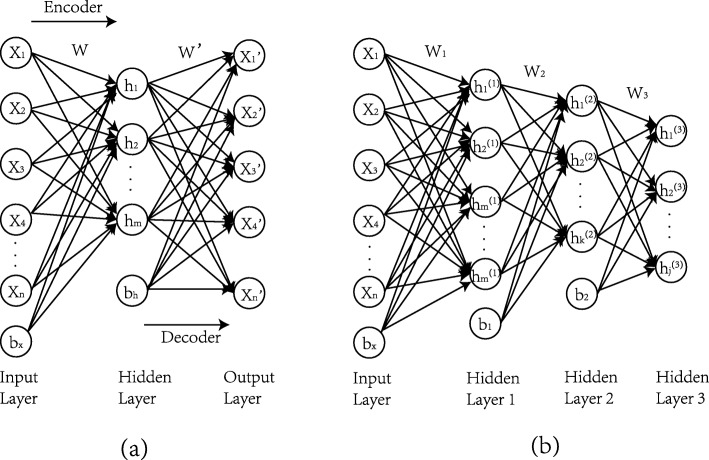
Architecture of autoencoder. **a** Structure of basic autoencoder. **b** Structure of three-layer stacked autoencoder

An autoencoder (AE) is an unsupervised method of dimensionality reduction and feature representation of raw data. Considering the *X*=*x*(1),*x*(2),*x*(3),...,*x*(*N*) be the training data set, Where *x*(*k*)*εR*^*n*^, *N* and *n* are the number of samples and features in the training data set. AE attempts to learn latent features which provide a better representation of the original data [43]. Suppose $H=\left \{h_{1}^{(l)},h_{2}^{(l)},h_{3}^{(l)},...,h_{m}^{(l)}\right \}$ is a set of hidden variables, and the main idea of AE is to learn the function *h*_*W, b*_(·), in which the targeted value is set to be equal to the input *h*_*W, b*_(*x*)=*x*. AE has two main parts known as an encoder and a decoder. Figure 4a shows the encoder section, where *x* data points are used as input for the AE and that are converted to a high level representation *h* by using the encoder function *f*(*W, b*_*x*_,*x*), where *W* and *b*_*x*_ are the parameters of the encoder function. In decoder section, the function *f*(*W*^′^,*b*_*h*_,*x*) tries to approximate the *x*^′^ of the raw input through the learned high-level representations, where *W*^′^ and *b*_*h*_ are the parameters of the decoder. Essentially, the goal of the AE training process is to find a set of optimal parameters (*W, W*^′^,*b*_*x*_,*b*_*h*_) by minimizing the difference between the given input to encoder “*x*” and reconstructed output by decoder “ *x*^′^”.

The difference of AE can be expressed in terms of a cost function. The formula is as follows.

2 $$\begin{array}{@{}rcl@{}} J_{AE}(\theta,x)=\frac{1}{N}\sum_{i=1}^{N}||x(i)-d_{\theta^{\prime}}(e_{\theta}(x(i)))||^{2}\\ +\lambda||W||_{2}^{2} \end{array} $$

The first term is the mean squared error (MSE) and the second term is the *L*2 regularization term to prevent overfitting. The *e*_*θ*_(·) refers to an encoder part having a parameter *θ*(*W, b*_*x*_) that transforms *x*∈*R*^*n*^ to represent *h*∈*R*^*m*^ according to the activation function *f*(*Wx*+*b*_*x*_). *W*∈*R*^*m*×*n*^ is the weight matrix of the encoder, and *b*_*x*_∈*R*^*m*^ is the bias term. Alike, $d_{\theta ^{\prime }}(\cdot)$ refers to an decoder part with the parameter *θ*^′^(*W*^′^,*b*_*h*_), which converts *h*∈*R*^*m*^ into *x*^′^∈*R*^*n*^ according to *f*(*W*^′^*h*+*b*_*h*_), where *W*∈*R*^*n*×*m*^ is the weight matrix of the decoder, and *b*_*h*_∈*R*^*n*^ is the bias term.

The training process of AE minimizes the difference error by using the gradient descent method to optimize the following.

3 $$\begin{array}{@{}rcl@{}} {arg min}_{\theta,\theta^{\prime}}J_{AE}(\theta,\theta^{\prime},x) \end{array} $$

The stacked autoencoder (SAE) consists of a multilayer autoencoder with the output of each hidden layer connected to the input of successive layers [47, 48]. The hidden variable for each layer provides a complex representation for the next layer in the SAE. For SAE, high-dimensional data is expected to obtain advanced features for downstream analysis [49, 50]. Figure 4b shows the structure of a SAE with 3 hidden layers. To make it simple, we have not shown the decoder part of the SAE at each layer. The hidden layers and hidden variables in each layer can be defined as needed.

### Deep flexible neural forest

A flexible neural tree (FNT) model was proposed by [40, 41], which solved the design problem of neural network structure. The tree structure optimization algorithm was used to select the model structure automatically. However, the structure of FNT was not useful to deal with multi-class problems [46, 51]. Increasing the depth of FNT can improve the performance of the model, but the cost of the parameter optimization algorithm increases. To solve above problem, a deep flexible neural forest (DFNForest) model was exploited to classify cancer subtypes [46].

**Fig. 5:**
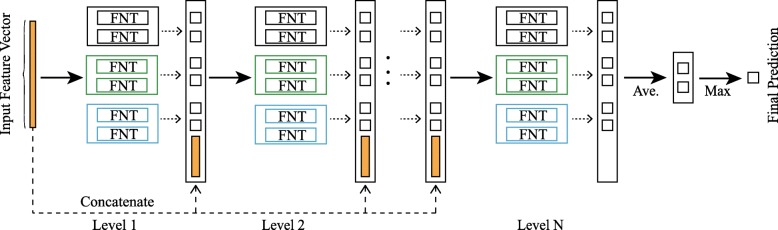
Illustration of the cascade forest structure. Three forests are generated by different grammar, the first forest (black) use function set F of {+_2_,+_3_, +_4_}, the second forest (green) use {+_2_,+_4_, +_5_}, and the last forest (blue) use function set F of {+_3_,+_4_, +_5_}

The cascade structure allows the depth of FNT to be increased without adding other parameters. As shown in Fig. 5, the cascade structure means processing features layer by layer, each layer can obtain new features, and the new features concatenated with the raw features are used as the input to next level. Although DFNForest model is inspired by deep forest [39], the base classifiers are different. Decision trees (DT) are used in deep forests, while FNT is used in DFNForest. We have proposed to use FNT as the base classifier instead of DT, because DT are not applicable to process continuous data, it is necessary to discretize continuous data first, which inevitably leads to information loss. The considered biological data are continuous data, so FNT is a better choice as a base classifier. The performance of ensemble learning is highly dependent on the accuracy and diversity of the base classifier. In order to ensure diversity, we have used different grammars to generate different FNT architectures. Suppose that three forests and two FNTs are used in each forest. As is illustrated in Fig. 5, the first forest uses function set F of {+_2_,+_3_, +_4_}, the second forest uses {+_2_,+_4_, +_5_}, and the last one uses {+_3_,+_4_, +_5_}. For each forest, M-ary method is used to convert multi-classification problem into multiple binary classification problems.

**Fig. 6:**
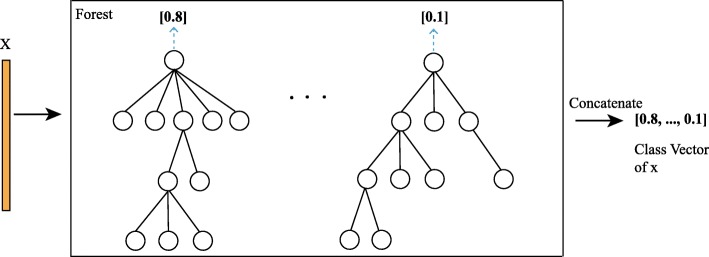
Illustration of class vector generation. Each FNT will generate an estimated value and then concatenated

Figure 6 shows the generation of class vectors in each forest. Given a sample, each FNT generates an estimated value. The estimated values of each FNT in a forest are concatenated as a class vector. The class vectors of all the forests in a layer are concatenated with raw input and considered as the input of the next layer. The entire data set is divided into three parts: training set, validation set, and test set. The validation set will verify the performance of the entire current cascade structure. When the accuracy does not change, the number of layers in the structure is determined. The number of cascade levels is automatically determined, which can be used for data set of different size.

The obvious advantage of DFNForest is the automatically design of the structure. The tree structure optimization algorithm automatically optimizes the FNT structure in each forest, and the cascade layers are adaptively determined, which can be used for data set of different size, especially for small-scale biological data. Moreover, the DFNForest model adopts the idea of ensemble learning, and the diversity and accuracy of the base FNT classifier can effectively improve the performance of our model.

### Hierarchical integration deep flexible neural forest framework

**Fig. 7:**
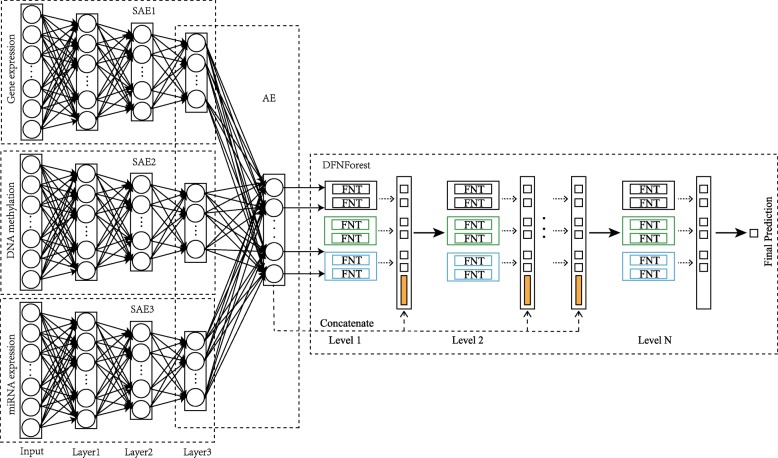
The hierarchical integration deep flexible neural forest framework

A hierarchical integration deep flexible neural forest framework is designed based on the SAE and DFNForest, named as HI-DFNForest, in which multi-omics data is integrated for cancer subtype classification. Data representations is learned respectively from each omics data using stacked autoencoders and all the learned representations are integrated into a layer of autoencoder to learn complex representations. Then the learned complex representations that are ultimately learned are used as the input to DFNForest model for cancer subtype classification. Figure 7 shows the hierarchical integration deep flexible neural forest framework. Three hidden layers in each SAE model are shown as an example to show our proposed hierarchical integration framework. Specifically, we use SAE models of different structures to learn the representation from gene expression, miRNA expression and DNA methylation data. Then learned representations are integrated into a layer of AE models to learn the complex representation. At last, the learned features are used as input to DFNForest model for cancer subtype classification.

The reason why we not only use gene expression information is that the cancer subtype classification is closely related to miRNA expression and DNA methylation [22–24]. There are two main challenges in integrating different omics data. First, multi-omics data usually come from multiple platforms, which usually have different representations and statistical properties. Second, multi-omics data for the same cancer is unlikely to be independent. Therefore, we propose a hierarchical integrated stacking autoencoder, which has the significant advantage of considering both intrinsic statistical properties of individual data and the correlation of different omics data. Given a sample, its input data consist of three omics data, gene expression, miRNA expression and DNA methylation data. Each omics data is passed through SAE with different structures, and the learned features are integrated into a layer of autoencoder to learn top-level representation. The final step is to use the previously learned features as input to the DFNForest classifier, which gives the final predicted label of the sample.

## Data Availability

The gene expression data, miRNA expression data and DNA methylation data can be downloaded from The Cancer Genome Atlas website at https://www.cancer.gov/about-nci/organization/ccg/research/structural-genomics/tcga/. The specific BRCA, GBM and OV datasets in our manuscript were available through https://github.com/tuiainao316/datasets.

## Abbreviations

BRCA: Breast invasive carcinoma
DFNForest: Deep flexible neural forest
DNN: deep neural networks
DT: Decision tree
FNT: Flexible neural tree
gcForest: multi-grained cascade forest
GBM: Glioblastoma multiforme
HI-DFNForest: Hierarchical integration deep flexible neural forest
KNN: K-nearest neighbor
NMF: Non-negative matrix factorization
OV: Ovarian cancer
PCA: Principal components analysis
RF: Random forest
SAE: Stacked autoencoder
SVM: Support vector machine
TCGA: The cancer genome atlas

## References

[1] Stingl J, Caldas C (2007). Molecular heterogeneity of breast carcinomas and the cancer stem cell hypothesis. Nat Rev Cancer.

[2] Dai X, Li T, Bai Z, Yang Y, Liu X, Zhan J, Shi B (2015). Breast cancer intrinsic subtype classification, clinical use and future trends. Am J Cancer Res.

[3] Liang M, Li Z, Chen T, Zeng J (2015). Integrative data analysis of multi-platform cancer data with a multimodal deep learning approach. IEEE/ACM Trans Comput Biol Bioinforma.

[4] West L, Vidwans SJ, Campbell NP, Shrager J, Simon GR, Bueno R, Dennis PA, Otterson GA, Salgia R (2012). A novel classification of lung cancer into molecular subtypes. Plos ONE.

[5] Prat A, Pineda E, Adamo B, Galván P, Fernández A, Gaba L, Díez M, Viladot M, Arance A, Muñoz M (2015). Clinical implications of the intrinsic molecular subtypes of breast cancer. Breast.

[6] Kim S (2012). Pathway-based classification of cancer subtypes. Biol Direct.

[7] Blanco-Calvo M, Concha Á, Figueroa A, Garrido F, Valladares-Ayerbes M (2015). Colorectal cancer classification and cell heterogeneity: A systems oncology approach. Int J Mol Sci.

[8] De-Shuang H, Chun-Hou Z (2006). Independent component analysis-based penalized discriminant method for tumor classification using gene expression data. Bioinformatics.

[9] Stratton MR, Campbell PJ, Futreal PA (2009). The cancer genome. Nature.

[10] Deng S-P, Zhu L, Huang D-S (2016). Predicting hub genes associated with cervical cancer through gene co-expression networks. IEEE/ACM Trans Comput Biol Bioinforma.

[11] Hanahan D, Weinberg RA (2011). Hallmarks of cancer: the next generation. Cell.

[12] Akbani R, Ng K-S, Werner HM, Zhang F, Ju Z, Liu W, Yang J-Y, Lu Y, Weinstein JN, Mills GB. A pan-cancer proteomic analysis of The Cancer Genome Atlas (TCGA) project. Cancer Research. 2014; 74(19):4262. Akbani R, Ng KS, Werner HM, Zhang F, Ju ZL, Liu WB, Yang JY, Lu YL, Weinstein JN, Mills GB. a pan-cancer proteomic analysis of the cancer genome atlas (TCGA) project. Cancer Research. 2014;74(19):4262.

[13] Weinstein JN, Collisson EA, Mills GB, Shaw KRM, Ozenberger BA, Ellrott K, Shmulevich I, Sander C, Stuart JM, Network CGAR (2013). The cancer genome atlas pan-cancer analysis project. Nat Genet.

[14] Shen S, Wang Y, Wang C, Wu YN, Xing Y (2016). Surviv for survival analysis of mrna isoform variation. Nat Commun.

[15] Sun D, Li A, Tang B, Wang M (2018). Integrating genomic data and pathological images to effectively predict breast cancer clinical outcome. Comput Methods Prog Biomed.

[16] Guo Y, Zheng J, Shang X, Li Z (2018). A similarity regression fusion model for integrating multi-omics data to identify cancer subtypes. Genes.

[17] Jahid MJ, Huang TH, Ruan J (2014). A personalized committee classification approach to improving prediction of breast cancer metastasis. Bioinformatics.

[18] Zheng C-H, Ng T-Y, Zhang L, Shiu C-K, Wang H-Q (2011). Tumor classification based on non-negative matrix factorization using gene expression data. IEEE Trans Nanobioscience.

[19] Marisa L, de Reyniès A, Duval A, Selves J, Gaub M. P, Vescovo L, Etienne-Grimaldi M-C, Schiappa R, Guenot D, Ayadi M (2013). Gene expression classification of colon cancer into molecular subtypes: characterization, validation, and prognostic value. PLoS Med.

[20] Leong HS, Galletta L, Etemadmoghadam D, George J, Study AOC, Köbel M, Ramus SJ, Bowtell D (2015). Efficient molecular subtype classification of high-grade serous ovarian cancer. J Pathol.

[21] Shang H, Jiang Z, Xu R, Wang D, Wu P, Chen Y (2019). The dynamic mechanism of a novel stochastic neural firing pattern observed in a real biological system. Cogn Syst Res.

[22] Bhattacharyya M, Nath J, Bandyopadhyay S (2015). Microrna signatures highlight new breast cancer subtypes. Gene.

[23] Bediaga N. G, Acha-Sagredo A, Guerra I, Viguri A, Albaina C, Diaz I. R, Rezola R, Alberdi M. J, Dopazo J, Montaner D (2010). Dna methylation epigenotypes in breast cancer molecular subtypes. Breast Cancer Res.

[24] Cantini L, Isella C, Petti C, Picco G, Chiola S, Ficarra E, Caselle M, Medico E (2015). Microrna–mrna interactions underlying colorectal cancer molecular subtypes. Nat Commun.

[25] Cimmino A, Calin GA, Fabbri M, Iorio MV, Ferracin M, Shimizu M, Wojcik SE, Aqeilan RI, Zupo S, Dono M (2005). mir-15 and mir-16 induce apoptosis by targeting bcl2. Proc Natl Acad Sci.

[26] Zhang W, Dahlberg JE, Tam W (2007). Micrornas in tumorigenesis: a primer. Am J Pathol.

[27] Chiou T-J, Aung K, Lin S-I, Wu C-C, Chiang S-F, Su C-L (2006). Regulation of phosphate homeostasis by microrna in arabidopsis. Plant Cell.

[28] Kim S, Park T, Kon M (2014). Cancer survival classification using integrated data sets and intermediate information. Artif Intell Med.

[29] Verhaak RG, Hoadley KA, Purdom E, Wang V, Qi Y, Wilkerson MD, Miller CR, Ding L, Golub T, Mesirov JP (2010). Integrated genomic analysis identifies clinically relevant subtypes of glioblastoma characterized by abnormalities in pdgfra, idh1, egfr, and nf1. Cancer Cell.

[30] Network CGA (2012). Comprehensive molecular portraits of human breast tumours. Nature.

[31] Network CGAR (2012). Comprehensive genomic characterization of squamous cell lung cancers. Nature.

[32] Network CGA (2012). Comprehensive molecular characterization of human colon and rectal cancer. Nature.

[33] Shen R, Olshen AB, Ladanyi M (2009). Integrative clustering of multiple genomic data types using a joint latent variable model with application to breast and lung cancer subtype analysis. Bioinformatics.

[34] Zhang S, Liu C-C, Li W, Shen H, Laird PW, Zhou XJ (2012). Discovery of multi-dimensional modules by integrative analysis of cancer genomic data. Nucleic Acids Res.

[35] Hartigan JA, Wong MA. J R Stat Soc Ser C (Appl Stat). 1979; 28(1):100–8.

[36] Ding C, He X. Cluster Structure of K-means Clustering via Principal Component Analysis. Lecture Notes in Computer Science. 2004; 46(4):414–418.

[37] Cireşan DC, Giusti A, Gambardella LM, Schmidhuber J. Mitosis detection in breast cancer histology images with deep neural networks. In: International Conference on Medical Image Computing and Computer-assisted Intervention. Springer: 2013. p. 411–8. 10.1007/978-3-642-40763-5_51 24579167

[38] Bao W, Huang Z, Yuan C-A, Huang D-S (2017). Pupylation sites prediction with ensemble classification model. Int J Data Min Bioinforma.

[39] Zhou Z-H, Feng J. Deep forest: Towards an alternative to deep neural networks. in Proc. 26th Int. Joint Conf. Artif. Intell; 2017, pp. 1–6.

[40] Chen Y, Yang B, Dong J, Abraham A (2005). Time-series forecasting using flexible neural tree model. Inf Sci.

[41] Chen Y, Yang B, Abraham A (2007). Flexible neural trees ensemble for stock index modeling. Neurocomputing.

[42] Vincent P, Larochelle H, Lajoie I, Bengio Y, Manzagol P-A (2010). Stacked denoising autoencoders: Learning useful representations in a deep network with a local denoising criterion. J Mach Learn Res.

[43] Ng A (2011). Sparse autoencoder. CS294A Lect Notes.

[44] Wang B, Mezlini AM, Demir F, Fiume M, Tu Z, Brudno M, Haibe-Kains B, Goldenberg A (2014). Similarity network fusion for aggregating data types on a genomic scale. Nat Methods.

[45] Rohart F, Gautier B, Singh A, Cao KAL (2017). mixomics: An r package for ‘omics feature selection and multiple data integration. Plos Comput Biol.

[46] Xu J, Wu P, Chen Y, Meng Q, Dawood H, Khan MM (2019). A novel deep flexible neural forest model for classification of cancer subtypes based on gene expression data. IEEE Access.

[47] Guo Y, Shang X, Li Z (2019). Identification of cancer subtypes by integrating multiple types of transcriptomics data with deep learning in breast cancer. Neurocomputing.

[48] Xu J, Xiang L, Liu Q, Gilmore H, Wu J, Tang J, Madabhushi A (2016). Stacked sparse autoencoder (ssae) for nuclei detection on breast cancer histopathology images. IEEE Trans Med Imaging.

[49] Ni L, Tian F, Ni Q, Yan Y, Zhang J (2019). An anonymous entropy-based location privacy protection scheme in mobile social networks. EURASIP J Wirel Commun Netw.

[50] Ni L, Zhang J, Jiang C, Yan C, Yu K (2017). Resource allocation strategy in fog computing based on priced timed petri nets. IEEE Int Things J.

[51] Wu P, Wang D (2018). Classification of DNA microarray for diagnosing cancer using a complex network based method. IEEE/ACM Trans Comput Biol Bioinforma.

